# A Critical Evaluation of Waste Incineration Plants in Wuhan (China) Based on Site Selection, Environmental Influence, Public Health and Public Participation

**DOI:** 10.3390/ijerph120707593

**Published:** 2015-07-08

**Authors:** Hui Hu, Xiang Li, Anh Dung Nguyen, Philip Kavan

**Affiliations:** 1 School of Economics and Management, Wuhan University, Wuhan 430072, China; E-Mail: lx2012@whu.edu.cn; 2 College of Arts & Social Sciences, the Australian National University, Acton, ACT 2601, Australia; E-Mail: anh.nguyen@anu.edu.au; 3 Faculty of Education, Science, Technology & Maths, University of Canberra, Bruce, ACT 2601, Australia; E-Mail: philip.kavan@canberra.edu.au

**Keywords:** waste incineration, multi-criterion assessment, game theory, site selection, environmental influence, public health, public participation

## Abstract

With the rapid development of the waste incineration industry in China, top priority has been given to the problem of pollution caused by waste incineration. This study is the first attempt to assess all the waste incineration plants in Wuhan, the only national key city in central China, in terms of environmental impact, site selection, public health and public participation. By using a multi-criterion assessment model for economic, social, public health and environmental effects, this study indicates these incineration plants are established without much consideration of the local residents’ health and environment. A location analysis is also applied and some influences of waste incineration plants are illustrated. This study further introduces a signaling game model to prove that public participation is a necessary condition for improving the environmental impact assessment and increasing total welfare of different interest groups in China. This study finally offers some corresponding recommendations for improving the environmental impact assessments of waste incineration projects.

## 1. Introduction

Municipal waste disposal is in great demand in China. The annual global waste disposal capacity growth rate has reached 8.42% while that of China is over 10% [1]. In effect, the whole world produces over 500 million tons of waste every year, with China contributing approximately 210 million tons to that number. In addition, since the early 1950s the accumulation of untreated municipal solid waste in China has amounted to over 7 billion metric tons [2]. Evidently, China’s municipal waste issue has been aggravated since the country promoted urbanization in the early 1990s [3]. In order to solve this problem, several important government departments in China, including the National Development and Reform Commission (NDRC) and the Ministry of Environmental Protection (MEP), have enacted related policies in support of waste incineration. With growing public concern over environmental issues, environmental protection in the context of waste disposal has become a crucial research subject in the process of realizing a sustainable development.

Waste incineration is a method of waste disposal whereby high temperatures are used to sufficiently oxidize the combustible components in waste. Compared with landfills and composting, incineration is more effective in dealing with municipal waste due to a few advantages, such as taking up comparatively small space, decreasing the volume of waste and generating electricity. Although there are bright prospects regarding the waste incineration industry, some issues, such as improper locations, lack of environmental impact assessments (EIA) and an excessive production of fly ash, have resulted from the fast development of waste incineration projects in China. Hence, it is necessary to ensure the process of waste incineration is harmless to the environment and public health.

As the only national key city in central China, Wuhan is confronted with the following serious problems in waste incineration projects: first, in terms of site selection, the illegal construction of waste incineration plants close to residential areas has endangered the residents’ health; second, as for the EIA, in which the public only has a limited participation, some governments, in collusion with waste incineration enterprises, manipulate the whole assessment process; third, from the perspective of pollutant emissions, some enterprises illegally dump and dispose of fly ash, which causes serious pollution to the environment and is a threat to public interests and public health; fourth, in terms of the technology used in incinerators, the older generations of incinerators are often much more dangerous to public health than more advanced incinerators—the advanced incinerators, used in Europe and some developed areas in China (like Shanghai and Shenzhen), have flue gas cleaning systems to reduce the air pollution [4], however, the incineration plants in Wuhan continue to use the old incinerators that produce a large amount of gas emissions. Finally, as for the supervision, the associated departments of the Wuhan Municipal Government are failing in their duty to supervise waste incineration under the pressure of disposing of excess garbage.

In the literature, campaigns against waste infrastructure have emerged since the 1970s in some developed countries, like the US, UK and France, because of the increasing public anxiety about the impacts of industrialism upon the environment and human health [5,6,7,8]. Similarly, the large-scale waste incineration in some Chinese cities, like Wuhan, has started to provoke public concerns and protests in recent years [9]. Since the waste incineration industry is experiencing a rapid growth, it is extremely necessary to find a proper way to address the problems mentioned above.

Existing studies have proposed some assessment frameworks for selecting waste incineration locations. For instance, Kermal and Erdagi [10], Xu *et al.* [11] and Pu [12] have established assessment frameworks based on some external influences. However, the previous studies have not applied a multi-criteria assessment model to analyze the waste incineration plant situation in China.

The present study is unique in both the research subject and its methods. First of all, the research focuses on Wuhan, which is the only national key city in central China and is a representative city experiencing rapid waste incineration development. Second, a multi-criterion analysis is established according to the literature to analyze all five waste incineration plants in Wuhan: Xinghuo, Xingou, Hankoubei, Changshankou and Guodingshan. By using the assessment model, the research analyzes the construction conditions of these plants from the perspectives of economy, society, public health and environment. A location analysis is also applied and some influences of waste incineration plants are illustrated. Moreover, a game theory model is introduced for further analysis to prove that the public participation is necessary for improving the EIA in this case. It also shows that the public participation can increase the total welfare of the different interest groups involved. On the basis of quantitative and qualitative analyses stated above, this study summarizes the strengths and weaknesses in China’s waste incineration industry and offers some suggestions for perfecting the Chinese EIA system.

## 2. Background Analysis

### 2.1. Production of Urban Solid Waste in China

According to data revealed by China’s National Bureau of Statistics [13,14,15,16,17,18,19], the statistics of collected and treated waste in China from 2007 to 2012 are shown in Table 1 below. Table 1 shows that the ratio of waste treated by incineration plants generally increased as more subsidies were provided to the incineration industry by the various Chinese government levels. By 2012, the ratio reached 19.7%, and there were 138 waste incineration plants in China. Currently, there is a growth tendency in their construction.

With the increasing amount of urban waste in China, the ratio of treatment rose gradually and crept up to over 80% in 2012. However, severe problems related to urban waste still exist with the rapid development of economy and urbanization. Cities face a serious situation of being surrounded by waste and it is difficult for the waste treatment ratio to reach the target (90%) set by China’s 12th Five-Year Plan (2011–2015) [14,20]. As a result, in the future, additional waste treatment facilities, including incineration plants, must be constructed.

### 2.2. BOT Model for Waste Disposal

Build-Operate-Transfer, known as BOT, refers to the mode whereby the government authorizes entities to raise money to design, build and operate a project before finally handing over the project to the government. Before the handover of the project, the BOT enables the project proponents to recover their investment and maintenance expenses as well as earn a satisfactory return on the investment. The BOT model, by introducing advanced technologies and management to effectively control the running costs, is a proper method to solve the government’s problems of insufficient funds. It is regarded as one of the most extensively applied modes in infrastructure projects in many countries.

**Table 1:** Statistics of municipal waste in China.

Year	Quantity of Municipal Waste (Million Metric Tons)	Ratio of Waste Treatment (%)	Landfilling			Incineration			Composting		
			Number of Plants for Wastes Treatment	Treatment Capacity (Metric Ton/Day)	Ratio* (%)	Number of Plants for Wastes Treatment	Treatment Capacity (Metric Ton/Day)	Ratio* (%)	Number of Plants for Wastes Treatment	Treatment Capacity (Metric Ton/Day)	Ratio* (%)
2007	15214.5	62	366	215179	81.5	66	44682	14.7	17	7890	3.8
2008	15437.7	66.8	407	253268	82.2	74	51606	14.9	14	5386	2.8
2009	15733.7	71.4	447	273498	80.4	93	71253	16.7	16	6979	2.9
2010	15804.8	77.9	498	289957	81.2	104	84940	16.9	11	5480	1.8
2011	16395.3	79.7	547	300195	80.8	109	94114	16.1	21	14810	3.1
2012	17080.9	84.8	540	310927	77.0	138	122649	19.7	23	12692	3.2

* Ratio = amount of certain waste treatment plants / amount of all waste treatment plants.

**Figure 1:**
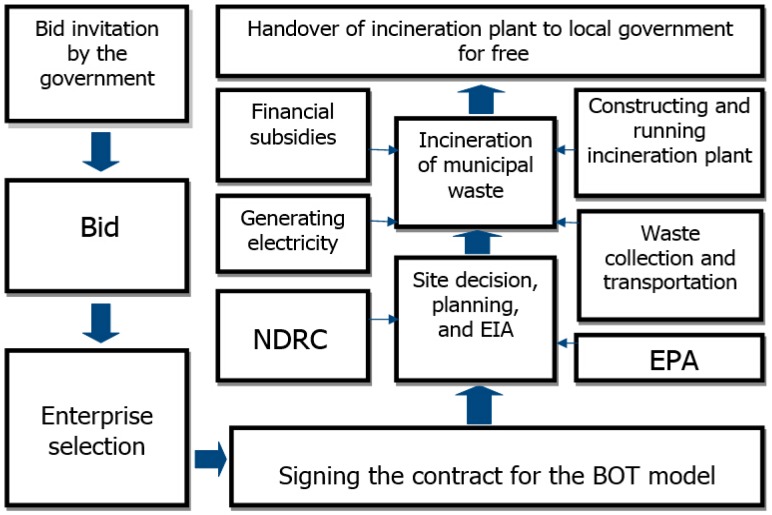
The waste incineration project based on the BOT mode.

The way how China deals with waste has been changed from the mode of governments being responsible for both the costs and profits of waste treatment in the planned economy to the BOT model in a market-oriented economy [21]. The BOT model is an effective way to improve environmental infrastructures in the cities of some developing countries. This is the way the incineration plants in Wuhan work. The application process of the BOT model in the constructions of incineration plants is illustrated in Figure 1.

First and foremost, the local government holds public tenders for the project and attracts firms to bid on it. After a firm’s tender for the project is accepted, the firm then starts to make plans for constructing the incineration plant based on the contract. This requires approval from the NDRC and the Environmental Protection Agency (EPA). Furthermore, the Bureau of Municipal Administration should be in charge of waste collection and its delivery to the plants. In the process of waste incineration, the firm can profit from the electricity generation as well as subsidies from the government. During the period of contract, the enterprises should be responsible for operating the plants and gaining operating revenue. Finally, the government becomes the owner of the plant for free when the contact expires.

### 2.3. General Situation of Waste Incineration Plants in Wuhan

Wuhan, the capital of Hubei Province, is the only national key city in central China. In 2007, the NDRC officially approved Wuhan with its satellite cities as a pilot area for establishing a resource-saving and environmental-friendly society. Hence, this present case study is of great importance to the sustainability of waste incineration in Wuhan.

There are five major waste incineration plants in Wuhan, which lie respectively in the Caidian, Dongxihu, Huangpi, Jiangxia and Qingshan districts. Their construction is of accordance to the plans put forward by the Wuhan Municipal Government. Those plants are located in the five major industrial areas of Wuhan and provide facilities of waste disposal in those areas. Table 2 lists the general information about the main incineration plants in Wuhan.

**Table 2:** General information of the incineration plants in Wuhan.

Name	Location	Time of Establishment	Daily Waste Disposal Capacity (Metric Tons)	Investment (Billion RMB)	Annual Electricity Production (Hundred Million Kilowatt Hour)	Parent Company
Xinghuo	Qingshan district	May, 2011	1000	4.52	1.2	Green Dynamic Co.
Xingou	Dongxi lake district	December, 2009	1000	4.07	0.9	Furlprotection Co.
Hankoubei	Huangpi district	January, 2009	2000	5.34	3.5	Green Fuel Co.
Changshankou	Jiangxia district	December, 2008	1000	3.01	1.6	Jingjiang Co.
Guodingshan	Hanyang district	December, 2006	1500	4.82	2.2	Borui Green Energy Co.

### 2.4. Policy Implementation and Plant Construction

In July 2013, the Hubei Environmental Protection Department reported that the Guodingshan waste incineration plant in the Hanyang district had seriously breached the environmental protection regulations. It operated without the EIA’s approval and did not have pollution control facilities. Moreover, there were thousands of residents living near the plant that suffered the air pollution from the waste incineration operation. The Hubei Environmental Protection Department notified that the plant should cease operation immediately, nonetheless, the Guodingshan waste incineration plant continues to operate without the government’s permission.

In this case, different levels of departments tried to shift their responsibilities. The EPA in the Hanyang District asserted that the waste incineration plant is beyond its supervision and the Wuhan EPA should take responsibility. Meanwhile, the Wuhan EPA criticized the local government in the Hanyang District for not removing the affected residents who live near the plant even after receiving 1.6 billion RMB in compensation for the residents’ removal. Additionally, the Urban Administration Bureau of Wuhan, which is in charge of waste collection and disposal, officially announced that it had stopped supplying waste to the Guodingshan waste incineration plant. However, the manager of the plant admitted that it is still disposing of municipal waste supplied by the Urban Administration Bureau of Wuhan. Therefore, apparently, we are faced with a series of contradictory statements made by the plant and the local governments.

The Wuhan Municipal Government faces a dilemma. According to its supervisory responsibilities the incineration plant should be shut down. However, once the incineration has been shut down, there will be no way to dispose of the waste in some districts such as Hanyang and Caidian. Therefore, there is a great deal of pressure on the government to either close the plants or allow the prohibited plants to operate.

**Figure 2:**
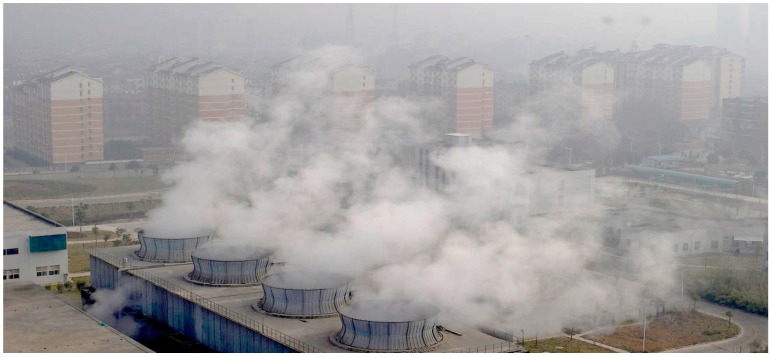
Guodingshan waste incineration plant with residential buildings nearby.

From the standpoint of interest groups, there are four groups involved in the process: the governments, corporations that are operating incineration plants, the EIAs and the residents. Based on game theory, the government is in the dominating position and firmly controls the decision-making. Various levels of governments in Wuhan pursue economic and political achievements unilaterally. They usually boost the GDP growth with investments in many fields, including waste incineration. Enterprises, in order to obtain maximum benefits, often collude with the EIAs and the governments to pass or even escape the EIA’s evaluation. As a result, a prohibited plant ends up emitting excessive waste gas which damages the public interest (see Figure 2).

The public is the weakest group, whose rights are also the easiest to be infringed. Some apartments are less than 100 meters away from the Guodingshan waste incineration plant, with thousands of residents living around it. The operation of the incineration plant poses great threats to the environment as well as the residents’ health. However, objections by the residents were largely ignored by the governments.

### 2.5. Inappropriate Site Selections of Waste Incineration Plants

**Figure 3:**
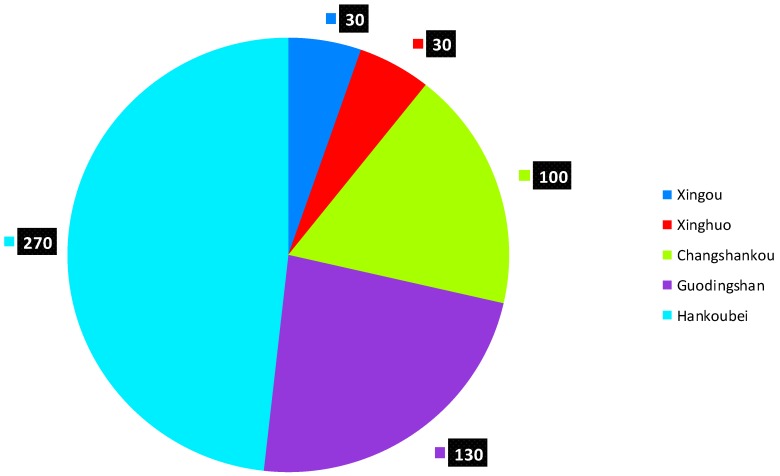
The output of fly ash in each incineration plant (metric ton/day).

**Figure 4:**
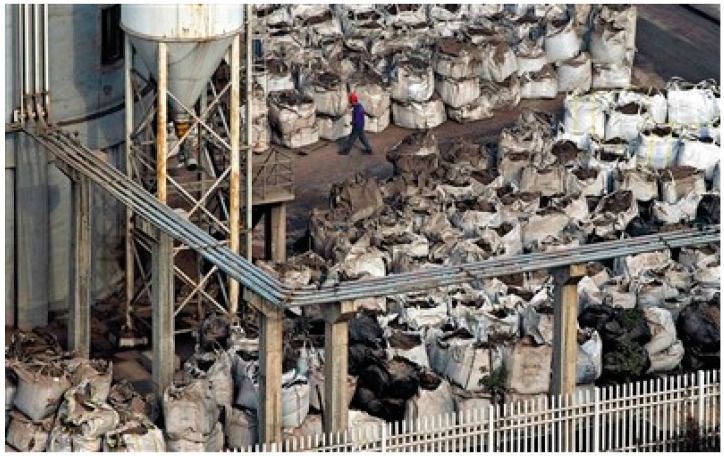
Fly ash in the open space of the Guodingshan waste incineration plant.

The present study has reviewed the waste incineration plant site selection process in Wuhan. The impropriety of the site selection can stated from four perspectives: first, the incineration plants in Wuhan are often built close to downtown areas, residential areas or even schools. The pollutants emitted by the plants may significantly damage the people’s health. For instance, a town center with a few schools, hospitals and residential areas is located 800 meters north of the Guodingshan waste incineration plant; Second, a few plants are built near lakes or rivers and one waste incineration plant is 500 meters away from one of Wuhan’s drinking water sources; third, there are not enough facilities near the plants which can deal with the waste incineration residues In addition, all five waste incineration plants in Wuhan have the same issue of illegally disposing of fly ash. The daily average output of fly ash from each plant is shown in Figure 3. Fly ash is a byproduct of waste incineration and contains dioxins. It is classified as a strong carcinogen by the International Agency for Research on Cancer. According to Chinese environmental protection law, fly ash residues are to be transported and disposed of only after it has been solidified in the incineration plants. However, all five waste incineration plants in Wuhan produce over 600 tons of fly ash each day and none of it receives any solidifying treatment (see Figure 3 and Figure 4).

If the fly ash is not appropriately solidified, the numerous dioxins produced may contaminate the air, water and soil. Because of the low water solubility and long biological half-life of dioxins, even small concentrations in water and soil can become concentrated in the food chain to develop into levels dangerous to human health. In other words, the untreated fly ash is a dangerous pollutant and could seriously damage the public health.

## 3. Methods and Analysis

### 3.1. The Multi-Criterion Evaluation Model for Site Selection

The present study, which is based on the literature, constructs an evaluation model involving various factors from the environment, economy, society and public health to analyze the incineration plant site selection process in Wuhan. The standards and references involved in the model can be found in Table 3.

According to these standards, the present study mainly selects a few groups of factors, principally economic, social, environmental and public health ones, to construct an evaluation model for assessing the incineration plants’ site selection. The multi-criterion assessment standards and the scores of various criteria for incineration plants are showed in Table 4 and Table 5. In the evaluation model, we use two different methods to standardize the data.

For the first method of standardization, the standardization value equal is [(x ‒ *β)* / (α ‒ *β*) / 2] + 1. For the second method of standardization, the larger raw data is, the smaller the fraction will be. Then the standardization value equals 3 / {[(x ‒ *β)* / (α ‒ *β*) / 2] + 1}. In these equations, x is the raw data, while *α* and *β* are the maximum and minimum values, respectively.

The present study analyzes the five incineration plants and evaluates each of them individually. For convenience, we use numbers from one to five to represent the Xinghuo, Xingou, Hankoubei, Changshankou and Guodingshan incineration plants, respectively.

It can be seen from Panel A in Table 5 that incineration plants 1, 2, 3 and 5 do not reach a passing score. In the present study, 60 is set as the passing score with the full score being 100. In panel B, all five incineration plants do not have high enough scores and only incineration plant 4 has a score (61.12) higher than the location assessment passing score. In Panels C and D, all incineration plants have higher scores than the passing score but some incineration plants have low scores in several sub-items. For example incineration plants 3, 4 and 5 have low scores for C1 which means these plants are close to residential buildings, schools or business districts. For panel E, the score of incineration plant 1 does not reach the passing score, meaning this plant significantly decreases the local residents’ total welfare. In sum, the locations of these plants do not meet all the standards of the EIA. Evidently, it is reasonable to conclude that incineration plants in Wuhan were established without much consideration of the local residents’ welfare nor the environment.

**Table 3:** Criteria of evaluation system with related references.

Criterion	Reference	Criterion	Reference
**Panel A: Environmental and Public Health Criteria**			
Distance from surface water	[22,23]	Land use suitability	[24,25,26]
Wetlands	[26,27,28]	Distance from water sources	[22,25,26,28,29,30,31,32,33,34,35,36]
Distance from residential areas	[22,23,25,28,30,31,37,38,39,40,41,42,43,44]	Traffic	[24,30,44,45]
Distance from flight paths	[23,24,26,31,33,39,40,44,46]	Distance from infrastructure and power lines	[25,30,39,40,41,44,47]
Rainfall	[39,47]	Air pollution index	[24,43,45]
Distance from railway	[38,39,40,43]	Odor	[25,44,45]
Floodplains	[25,26,30,36,39,40,44,47]	Distance from natural springs	[44]
Distance from irrigational canals	[38]	Distance from highway	[26,39,40]
Distance from forest lands	[30,31,46]	Distance from tourism areas	[44]
Ecological impacts	[28,44]	Distance from leisure areas	[26,39]
Distance from archaeological sites	[23,25,30,33,36,38,39,41,44]	Distance from burial yards	[44]
Distance from other special areas	[30]	Noise	[45]
		Dust	[44]
**Panel B: Economic Criteria**			
Property	[44]	Price of land	[23,30,36,37,39,42,46]
Land availability	[39]	Proximity to power lines	[25]
Haul distance	[23,24,28,30,39,43,44,46]	Transportation costs	[25,27,34,39,44]
Distance from roads	[22,23,25,30,31,36,37,38,39,40,41,43,44,46]	Distance from industrial areas	[22,31,39,44]
Proximity to infrastructure	[25]	Final usage suitability	[44]
**Panel C: Social Criteria**			
Approval of local residents	[24,29,44]	Political concern	[24,29]
Risk perception	[24,28,45,47]	Public reaction	[27]
Heritage	[29]	Local development	[36,45]
Labor	[24,27,32]		

**Table 4:** The multi-criterion assessment.

Item	Classification	Rank	Rank Data for Incineration Plant				
			1	2	3	4	5
**Panel A: Cost**							
A1. Cost of construction and trash transportation	Computed by the standardization of construction and trash transportation cost (applied to the second standardization)	—	4.52	4.07	5.34	3.01	4.82
**Panel B: Location of Incineration Plants**							
B1. Difficulty in obtaining land	Computed by the standardization of lowly-used and non-used land proportion in the area around incineration plant (applied to the first standardization)	—	20	50	5	50	10
B2. Condition of trash transportation road	a. Well-facilitated (width between 15–24 m)	3	2	1	1	3	3
	b. Ordinarily-facilitated (width between 8–14 m)	2					
	c. Poorly-facilitated (width narrower than 8 m)	1					
B3. Relationship with affiliated facilities	a. Facilities within a 15 km radius of the plants	3	1	1	1	1	1
	b. Facilities beyond a 15 km radius of the plants	2					
	c. No affiliated facilities	1					
B4. Relationship with other municipal projects and facilities nearby	a. Well-compatible (near sewage treatment facilities)	3	1	1	1	1	1
	b. Ordinarily-compatible (no projects or facilities nearby)	2					
	c. Poorly-compatible (near residential areas or schools)	1					
**Panel C: Impact on Surroundings**							
C1. Impact on the usage of land influenced	Computed by the standardization of current area influenced by residential buildings, schools and business districts, etc. (applied to the second standardization)	—	30.01	15.32	50.79	40.26	65.48
C2. Impact on local historical sites	a. No historical sites within a 500 m radius	3	3	3	3	3	3
	b. Provincial historical sites within a 500 m radius	2					
	c. National historical sites within a 500 m radius	1					
C3. Impact on local scenic spots	Computed by the standardization of influenced area of artificial and natural landscapes (applied to the second standardization)	—	5.23	1.25	1.05	1.56	1.85
**Panel D: Impact on Environment**							
D1. Impact on land ecosystem	Computed by the standardization of influenced wetland, forest and other important reservation area (applied to the second standardization)	—	0.04	0.01	0.02	0.01	0.01
D2. Impact on water ecosystem	Computed by the state of lake area within a 5 km radius around incinerators	—	40.42	10.25	30.56	13.15	15.26
D3. Air pollution	a. Located near lake area	3	3	3	3	2	3
	b. Located in the flatland between lake and mountain area	2					
	c. Located near mountain area, but far from lake area	1					
**Panel E: Impact on Local Residents**								
E1. Impact of incineration plants’ construction and operation on local residents	Computed by the standardization of residents influenced (applied to the second standardization)	—	529407	28503	40285	31840	72037
E2. Impact of waste transportation towards local residents	Computed by the standardization of residents living near main roads influenced.	—	732	602	1296	890	2380
E3. Impact on local traffic	a. High level of current traffic service	3	1	2	2	3	3
	b. Medium level of current traffic service	2					
	c. Low level of current traffic service	1					

NOTE: “—” means it is not applicable.

**Table 5:** The scores of various criteria.

Item	Weight (%)	Score for Waste Incineration Plant				
		1	2	3	4	5
A1	100	1.30	1.56	1	3	1.17
Total score in panel A	100	43.33	52	33.33	100	39
B1	22.82	1.66	3	1	3	1.22
B2	18.87	2	1	1	3	3
B3	21.03	1	1	1	1	1
B4	37.29	1	1	1	1	1
Total score in panel B	100	44.64	48.55	33.33	61.12	47.58
C1	44.11	1.89	3	1.24	1.50	1
C2	33.60	3	3	3	3	3
C3	22.29	1	2.73	3	2.40	2.16
Total score in panel C	100	68.82	97.99	74.12	73.49	64.36
D1	35.759	1	3	1.50	2.02	2.48
D2	21.221	1	3	1.27	2.51	2.25
D3	43.03	3	3	3	2	3
Total score in panel D	100	62.02	100	69.895	70.51	88.50
E1	45.73	1	3	2.86	2.96	2.55
E2	30.19	2.61	3	1.68	2.26	1
E3	24.08	1	2	2	3	3
Total score in panel E	100	49.54	91.97	76.55	91.94	73.01

NOTE: Total score in panel x = the ratio of calculated value to the full value (300) of each panel × 100.

### 3.2. The Location Analysis of the Guodingshan Incineration Plant

**Figure 5:**
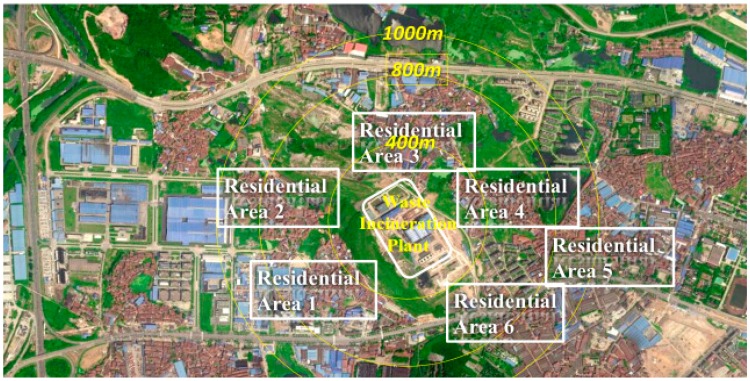
The location analysis of Guodingshan waste incineration plant.

The Guodingshan municipal waste incineration plant is surrounded by several residential areas. This is prohibited by national standards—Pollution Control Standards for Hazardous Wastes Incineration. According to the standards, the safe distance for waste incineration was set as 1000 meters before 2009. After 2009, it was adjusted to 800 meters, but the safe distance for the Guodingshan waste incineration plant was reduced to 400 meters by the local bureau of environmental protection. In Figure 5, we show three concentric circles which represent 400 meters, 800 meters and 1000 meters away from the waste incineration plant.

Circle 1: with a radius of 400 meters

In residential area 3, half of the residents (over one thousand people) have moved away because they cannot tolerate the harmful odors. In this residential area, 33 people have developed respiratory cancer. The air pollution caused by waste incineration places the residents at serious risk of respiratory disease.

Circle 2: with a radius of 800 meters

It is regulated that the distance from the incineration plants to public facilities such as schools should be further than 800 meters. However, there are two kindergartens and one primary school within this area. There are over 30,000 residents living in this area.

Circle 3: with a radius of 1000 meters

The Qinduankou water plant is located in this area, which supplies the Hanyang district with water. Once the water is polluted by the flying ash emitted by the incineration plants, the dioxins would be hazardous to people’s health when they drink the contaminated water.

### 3.3. The Game Theory Analysis of the EIA

Besides the multi-criterion evaluation model and location analysis, this study aims to reveal the important factors which influence the location selection of incinerators. As for the social factors, we use the game theory to analyze the role that the public play in the process of EIA. The game theory model in this study has four sides: the government (G), the enterprise (E), the public (P) and the EIA organization (O). Accordingly, we compare the aggregated revenue before the public participation and after. In the model, σ*_G_*, σ*_E_* and σ*o* are the probability functions representing the reactions from the government, the enterprise and the EIA organization (signal receivers) respectively. *µ*_G_, *µ*_E_ and *µ*_O_ are the expected benefit of the government, the enterprise and the EIA organization.

When the public (P) participation is included, we use a perfect Bayesian model equilibrium to deduce our conclusion. In this model, the sender signal used to protect the environment is the public (*p*_1_) who prefers environmental protection. The reactions from the public (signal sender) can be represented as a probability function σ*_P_*. The expected benefit for the government, the enterprise and the EIA organization are *µ'_G_*, *µ'_E_* and *µ'_O_*. We use superscript prime to represent the revenue with the public participation. This study compares the costs with and without the public participation:

$$∆µ={{µ}^{\prime }}_{sum}-{µ}_{sum}=({{µ}^{\prime }}_{P}-{µ}_{P})+({{µ}^{\prime }}_{G}-{µ}_{G})+({{µ}^{\prime }}_{E}-{µ}_{E})+({{µ}^{\prime }}_{O}-{µ}_{O})\approx {{µ}^{\prime }}_{P}-{µ}_{P}={{µ}^{\prime }}_{P}-0={{µ}^{\prime }}_{P}(\sigma_{\text{P}},\sigma_{\text{G}},\sigma_{\text{E}},\sigma_{\text{O}},p_{1})≻0$$

Without the public participation, *µ_P_* equals zero approximately. Thus the variation of profit for the whole group is obviously positive because *µ′_P_* is greater than 0. Taking the costs into consideration, we have:

$$∆µ-∆C={{µ}^{\prime }}_{P}-∆C={{µ}^{\prime }}_{P}\theta ({C^{\prime }}_{p}{C^{\prime }}_{g}{C^{\prime }}_{e}{C^{\prime }}_{o})-({C^{\prime }}_{p}-{C^{\prime }}_{e}-{C^{\prime }}_{o}-{C^{\prime }}_{g})-(C_{p}-C_{e}-C_{o}-{C^{\prime }}_{g})≻0$$

In the above equations, *θ* is the total coefficient of the strategy probability function σ, which is the function of cost (C). Since *θ* is positive, the first part, *µ′_P_θ(C′_p_C′_g_C′_e_C′_o_)*, is also positive. The cost of the public is less than the total cost of the other three sides regardless of whether the public participates or not. Both the second part (*C'_p_* − *C'_e_* − *C'_o_* − *C'_g_*) and the third part (*C_p_* − *C_e_* − *C_o_* − *C_g_*) are negative. Hence, ∆*µ −* ∆*C* should be positive. In other words, public participation can increase the aggregated revenue (profit minus cost) of the four sides.

With public participation, the cost and the profit increase compared to the cost and profit respectively without the public participation. In addition, the increment of cost is smaller than the increment of the benefit owing to the promotion of the environmental quality and public health which increase the total welfare of the four interest groups.

According to these analyses, the EIA organization, the government and enterprises can easily collude, which maintains a balance between the economic interests and environmental protection. Therefore, public participation should be introduced into the EIA in China.

## 4. Results and Discussion

### 4.1. Solutions from the Perspective of the EIA

With the above analyses using the models related to the EIA, we find that the waste-incinerating enterprises only aim to maximize their own profits instead of environmental and public health benefits. The EIA system in China is usually not restrictive. The Chinese Environmental Impact Assessment Act stipulates that “*if a construction project has not passed the EIA, the associated government departments should not give permission for the project and construction cannot start*” [33,48]. However, the Guodingshan waste incineration plant, which severely violated the Environmental Impact Assessment Act in China, was established before the EIA [49]. In addition, the EIA did not give any positive feedback for establishing this plant. According to the analyses above, it can be concluded that those incineration plants in Wuhan are established with less public involvement. These investment projects take only the government’s achievements and the investors’ profits into account and completely overlook environmental issues, public health and interests. To solve this problem, China should further improve the related regulations of the EIA:

1. The implementation of regulations still has a long way to go in China. Governments have not done a good job on enforcing compliance with the regulations. The non-compliant behavior is usually due to corruption and resource limitations facing the government. Hence, besides improving the laws and regulations associated with the EIA, promoting transparency in administration and fostering accountability at all governmental levels is crucial for fighting corruption and enforcing compliance with the regulations. In the present study, the waste-incinerating power plants and Wuhan Municipal Government have severely violated regulations and laws. Within China’s criminal law, illegally discharging, dumping and disposing over three tons of hazardous wastes can be identified as severely polluting the environment, which would be subject to criminal prosecution. The discharge of large amount of hazardous waste every day by the five plants in Wuhan should be a serious crime.
2. In terms of protecting the public health, improving the relevant techniques and standards of the EIA is a necessity. The newly-revised Standard for Municipal Solid Waste Incineration raised the dioxins emission standard [50]. This revision accelerates the upgrading of waste incinerators which do not meet the standard. Meanwhile, the introduction and development of more eco-friendly waste-incinerating techniques promotes the efficiency of incinerators and plays a vital role in reducing fly ash.
3. The government should improve the evaluation criterion in the waste-incinerating field and let the EIA become more independent. It is critical to eliminate common interests between the EIA organizations and incineration plants. Consequently, a large amount of inefficient waste disposal capacity can be phased out. This change is unquestionably beneficial to the environment.
4. Governments could implement market-based measures to provide incentives to waste incineration plants and protect public interests. These measures include subsidies for applying eco-friendly techniques, tradable pollution permits, pollution insurance and environmental liability insurance [51,52].

### 4.2. Measures for Improving the EIA and Its Supervision

It is widely criticized that Chinese governments lack independence in organizing and supervising waste disposal. On the one hand, the government shoulders responsibilities for waste disposal and has the responsibilities of evaluating and supervising incineration projects. On the other hand, the government is an economic entity in the BOT model and has economic and political interests in running incineration projects.

There is also a clear relationship between the governments and the incineration projects. The local government provides the incineration plants 50 to 140 RMB for each metric ton of disposed waste in the name of subsidizing renewable energy projects. Establishing incineration plants is financially supported by the Wuhan Municipal Government. This study suggests that measures should be taken to make the governments play a more effective role in environmental protection and supervision:

1. In China, local officials in governments have devoted tremendous amounts of attention to enhancing regional economic growth because of the economic performance-based promotion scheme. It is necessary to improve the scheme and make environmental conditions a key factor in the appraisal of officials.
2. Governments and the EPA should be independently in charge of environmental protection and supervision but no longer be involved in investing in incineration plants. In China’s current political system, the supervisory function of the Chinese People’s Political Consultative Conference and environmental non-government organizations need to be reinforced and they can be given an oversight role in environmental protection.
3. The data and information revealed by the EPA has to be open to ordinary people. It is also essential for government to have direct communication with the public. All of these could muster support from local residents for the construction of waste incineration plants in China [53,54]. For this case in Wuhan, formal public participation is not a part of the EIA system currently. Some forums and provisions (like public and non-government organizations) for the public and consultation could be useful for enhancing public participation. In terms of the revelation of real environmental information to the public, the new open government information legislation has entered into force in China. The public could invoke the law to request agencies to disclose environmental data and information.
4. Site selection for the waste incineration plant is a key factor influencing not only public health but also the environment. For the local residents, incineration plants are public facilities which can benefit the whole society and the environment. Nevertheless, nobody wishes to live next to a waste incineration plant without any compensation lest his or her health be damaged [55].

The present research shows that the government needs to select the sites of incineration plants carefully. The final decision for locations has to be made using a multi-criterion assessment and in accordance to most residents’ attitudes. The academics and scientists, who can independently make judgement on their expertise, should be in charge of the multi-criterion assessment. In terms of most cases in China, if the process of multi-criterion assessment is generally open, independent and transparent, the residents’ attitudes could be influenced by the scientific assessment.

## 5. Conclusions

The present study focuses on incineration plants in Wuhan—the only national key city in central China. Apparently, the waste incineration plants bring about both economic (e.g., increasing GDP) and environmental (e.g., environmental protection) achievements for the local governments. Under the abnormal GDP-oriented evaluation system in China’s political system, the government is likely to unilaterally pursue economic achievements regardless of public health and environmental issues [5,56]. In that case, the local governments in Wuhan tend to promote the establishment of waste incineration plants and sometimes even help them to escape the EIA.

In order to evaluate these waste incineration plants’ environmental impact, this study constructed an analytical framework to explore related environmental, economic, social, and public health influences of waste incineration plants. It also, based on the framework, created a quantified multi-criterion EIA model and a location analysis for evaluating these plants in Wuhan. The results show that these incineration plants do not meet standards of the EIA and they could be harmful to the environment and health of the residents living around these plants.

Additionally, the present study used a game theory model to analyze the relationships among the four participants of incineration plants. It is revealed that the EIA organization, local governments and enterprises can easily collude. Hence, public participation can play an effective role in the supervision of incineration plants. According to these results, this study suggested to improve the systems and related regulations of the EIA. It is necessary to establish an effective mechanism to make the governments and environmental non-government organizations play a more effective role in environmental protection and supervision.
